# Hypersonic Aerodynamic Force Balance Using Micromachined All-Fiber Fabry–Pérot Interferometric Strain Gauges

**DOI:** 10.3390/mi10050316

**Published:** 2019-05-11

**Authors:** Huacheng Qiu, Fu Min, Yanguang Yang, Zengling Ran, Jinxin Duan

**Affiliations:** 1 China Aerodynamics Research and Development Center, Hypervelocity Aerodynamics Institute, Mianyang 621000, Sichuan Province, China; huacheng.qiu@outlook.com (H.Q.); minfu@cardc.cn (F.M.); daybreakdjx@126.com (J.D.); 2 University of Electronic Science and Technology of China, Key Lab of Optical Fiber Sensing and Communications, Chengdu 610000, Sichuan Province, China; ranzl@126.com

**Keywords:** hypersonic wind tunnel, aerodynamic force balance, all-fiber Fabry–Pérot interferometer, strain gauge, temperature self-compensation

## Abstract

This paper presents high-sensitivity, micromachined all-fiber Fabry–Pérot interferometric (FFPI) strain gauges and their integration in a force balance for hypersonic aerodynamic measurements. The FFPI strain gauge has a short Fabry–Pérot cavity fabricated using an excimer laser etching process, and the deformation of the cavity is detected by a white-light optical phase demodulator. A three-component force balance, using the proposed FFPI gauges as sensing elements, was fabricated, calibrated, and experimentally evaluated. To reduce thermal output of the balance, a simple and effective self-temperature compensation solution, without external temperature sensors, is proposed and examined through both oven heating and wind tunnel runs. As a result of this approach, researchers are able to use the balance continuously throughout a wide range of temperatures. During preliminary testing in a hypersonic wind tunnel with a free stream Mach number of 12, the measurement accuracies of the balance were clearly improved after applying the temperature self-compensation.

## 1. Introduction

Free-space Fabry–Pérot (FP) interferometers are widely employed for applications in lasers, spectroscopy, and filters, just to name a few. There are different types of FP structures, where the stable optical interferometer consisting of two mirrors on either side of an optically transparent medium (cavity) is most often used. Compared with conventional free-space FP devices, the development of an all-fiber FP interferometric (FFPI) device represents a big step, not only in terms of great miniaturization but also in terms of the enrichment of the various structures. The basic idea of using a single fiber to realize a fiber-optic FP cavity was presented by Cielo [1] and by Yoshino et al. [2]. Petuchowski et al. [3] discussed the implementation of a FFPI device with fiber ends to serve as mirrors. On account of the advantages, such as capability of responding to a wide variety of parameters, high resolution, and miniature size, FFPI sensors were demonstrated to be especially attractive for the measurement of numerous physical and chemical parameters in past years [4,5,6,7,8].

Furthermore, the FFPI sensors are physically separated from the laser and electrical signal and, therefore, are robust to extreme environments such as high vacuum, elevated temperature, and magnetic field, making these types of sensors well suited for usage in hypersonic wind tunnel experiments. The underlying purpose of wind tunnel testing is to understand the performance characteristics of an aircraft, in an environment that closely simulates “true” flight conditions. A force balance is a precision-machined instrument that is capable of accurately and precisely measuring the aerodynamic loads imparted on the aircraft model, by applying strain gauges at strategic locations to measure the strain due to applied loads. Owing to the excellent characteristics of fiber optical sensors, several researches [9,10,11,12,13,14] reported designing and fabricating wind tunnel balances based on FP interferometric (FPI) or fiber Bragg grating (FBG) sensors, for applications in a harsh hypersonic tunnel environment, to assess the benefits of improved accuracy, higher stiffness, increased resolution, or thermal stability. Typical strain resolution for an FPI sensor is approximately 0.15 micro strain (με) [15]. Newer FBG sensors report a strain resolution of 0.4 με [16]. Edwards [13] noted better accuracy (0.8%) and resolution (0.002%) of an FPI sensor directly compared to a foil strain gauge sensor, which is normally used in conventional wind tunnel balances.

One main drawback of the FFPI sensors, just like all strain gauges, is the cross sensitivity to temperature due to thermal expansion of its base material. Since FFPI is an air cavity, its optical path difference is quite insensitive to temperature due to the low thermal expansion coefficient of the fiber and thermo-optical coefficient of the air cavity, endowing itself a minimal temperature strain cross sensitivity. However, in field strain tests, thermal expansion of the base material could introduce a large thermal strain when there are evident temperature variations, which consequently degrade the accuracy of the effective strain measurements [17,18,19]. In wind tunnel simulated hypersonic flow with Mach number ≥10, the flow medium needs to be heated to elevated temperatures up to 600 K or above [19] and this results in heating of the aircraft model and, hence, the balance and strain gauges located inside. Temperature influence becomes an important issue, and compensation is necessary in this case. A traditional way to address this problem is to use a separate temperature sensor to obtain the temperature information and, thereafter, compensate for the temperature effect in the strain readings. In wind tunnel applications, however, this appeared as very unreliable and unrepeatable [20,21,22,23].

This paper presents a high-sensitivity, micromachined FFPI strain gauge and its integration in a force balance for hypersonic aerodynamic measurements. The gauge has a short Fabry–Pérot cavity fabricated using excimer laser processing, and the deformation of the cavity is detected by a white-light optical phase demodulator. A three-component wind tunnel balance, using the proposed FFPI gauges as sensing elements, is fabricated, calibrated, and experimentally evaluated. To reduce the thermal output of the balance, a simple and effective temperature self-compensation solution, without external temperature sensors, is proposed and examined through both oven heating and wind tunnel runs. As a result of this approach, researchers are able to use the balance continuously throughout a wide range of temperatures. During preliminary tests in a hypersonic wind tunnel, the measurement accuracies of the balance clearly improved after applying the proposed self-temperature compensation.

## 2. Principle of the FFPI Strain Gauge

### 2.1. Structure and Theoretical Analysis

**Figure 1:**
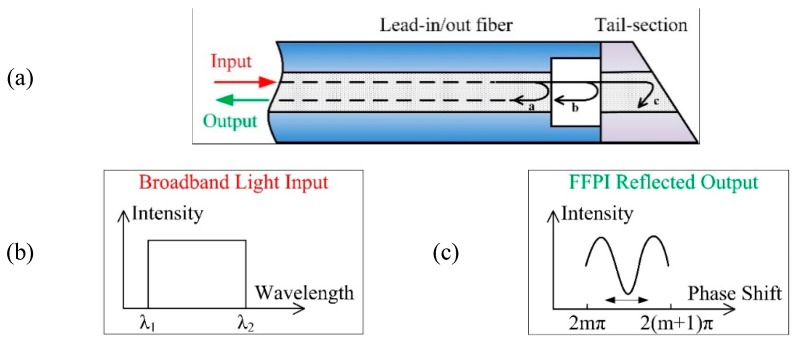
Concept of the proposed fiber Fabry–Pérot interferometric (FFPI) strain gauge, (**a**) design scheme, (**b**) input light spectrum, and (**c**) reflected output light spectrum.

The structure design of the proposed FFPI strain gauge is shown in Figure 1a. This gauge is composed of two fibers spliced together, with a lead-in/out fiber on one side and a tail-section with an arbitrary length on the other side (see Figure 1a). The FP cavity is formed by using an air hole as the reflective element. The tail of the fiber is cut with an oblique angle, to avoid any additional interference.

When a broadband light with a wide wavelength spectrum (1550–1600 nm) is transmitted through the FFPI, as shown in Figure 1b, the intensity of the reflected light *I_r_* at a particular wavelength depends on the distance between the two reflecting surfaces, *d*, according to the Airy function [24].

(1) $$I_{r}=\frac{4R\sin^{2}\left(\phi /2\right)}{{\left(1-R\right)}^{2}+4R\sin^{2}\left(\phi /2\right)}I_{i}$$

where *R* is the reflectivity of the mirrors, and *I_i_* is the intensity of incident light; ϕ is the propagation phase shift in the interferometer, which is calculated by

(2) ϕ=4πndλ+ϕ0

where *λ* is the wavelength of incident light, *n* specifies the order of interference, and $\phi_{0}$ is the initial phase.

The light from the light source is propagated along the lead-in/out fiber to the sensor head which is a low-finesse Fabry–Pérot interferometer formed by the end-faces of the lead-in/out fiber and a reflecting fiber. A fraction of the incident light (labeled as *a* in Figure 1a) is firstly reflected (reflectance *R* ≈ 4% results in the intensity *I*_1*r*_ ≈ 4% *I_i_*) at the end-face of the lead-in/out fiber and returns down the fiber, forming the reference beam. The transmitted light passes through the air gap and is reflected back. The reflected light *b* (*I*_2*r*_ ≈ 3.69% *I_i_*) is recoupled into the lead-in/out fiber and interferes with the reference beam. After the third reflection, the intensity *I*_3*r*_ (≈ 0.15% *I_i_*) is much lower than in the first two; thus, the influence of *I*_3*r*_ can be neglected. It means that it is enough to consider only two reflected beams.

The intensity of the reflected light *I_r_* from Equation (1) can then be simplified as

(3) $$I_{r}=I_{1}+I_{2}+2\sqrt{I_{1}I_{2}}\cos\phi .$$

The typical optical spectrum of the two-beam interference (see Figure 1c) is similar to a sinusoidal function, according to Equation (3). When ϕ=2mπ (*m* = 0, 1, 2, …), the interference intensity becomes maximum and, if ϕ=2(m+1)π, the interference intensity becomes minimum. As ϕ relies on the characteristics of the cavity (the cavity length changes), the optical spectrum will be shifted and can be used for strain sensing.

### 2.2. Gauge Fabrication

**Figure 2:**
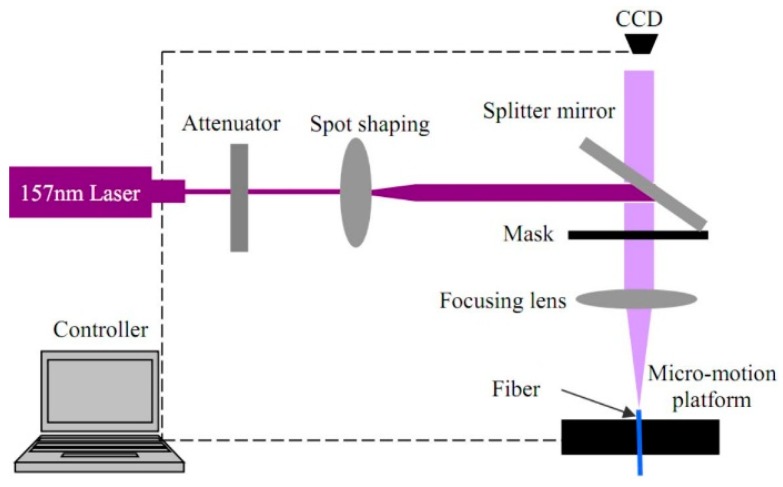
Schematic of micromaching system based on 157-nm excimer laser.

The gauges used in this work were fabricated using an excimer laser processing (shown in Figure 2). The optical absorption coefficient at 157 nm has a high value of up to ~20,000 cm^−1^, making it possible to achieve high-quality cool machining of silica fibers. The 157-nm laser micromachining system consists of a 157-nm pulsed laser, an optical focusing system with 25× demagnification, and a precise translation stage used to mount the fiber to be engraved. A comprehensive description of the system can be found in Reference [25].

**Figure 3:**
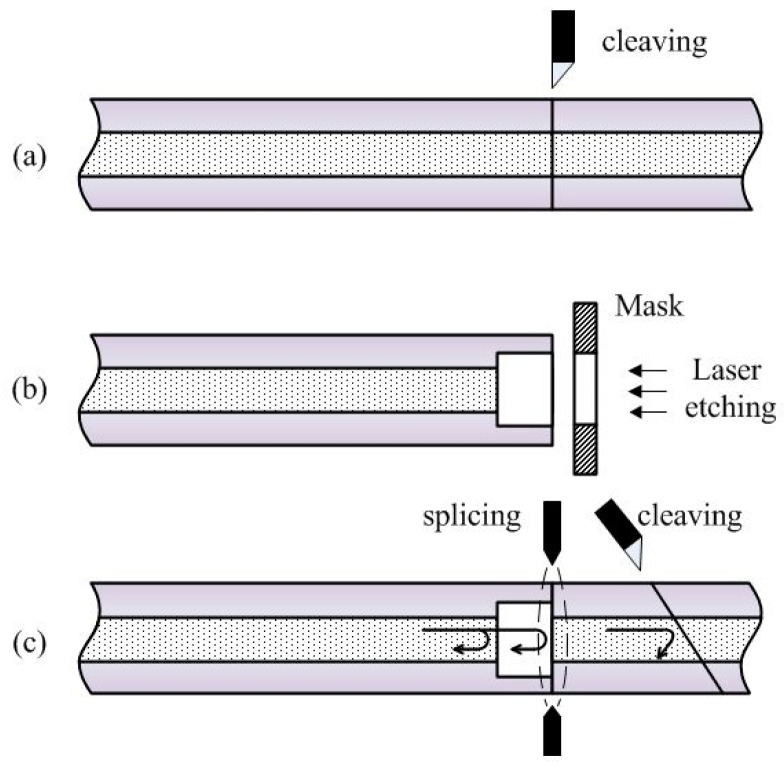
Fabrication process.

The manufacturing process is straightforward, as shown in Figure 3. During the first phase (Figure 3a), cleaving is performed to trim the sensor-forming surface. The cleaved fiber is further etched using a 157-nm-wavelength ultraviolet laser with predetermined parameters, such as laser power density, pulse duration, frequency, and number (Figure 3b). The last phase of micromachining (Figure 3c) encompasses fusion-splicing of the etched and cleaved fibers, in order to obtain an air cavity. Moreover, one end of the fused fiber was cut to a distance much longer than the cavity depth with an oblique angle, to avoid any additional interference at the tail of the fiber.

**Figure 4:**
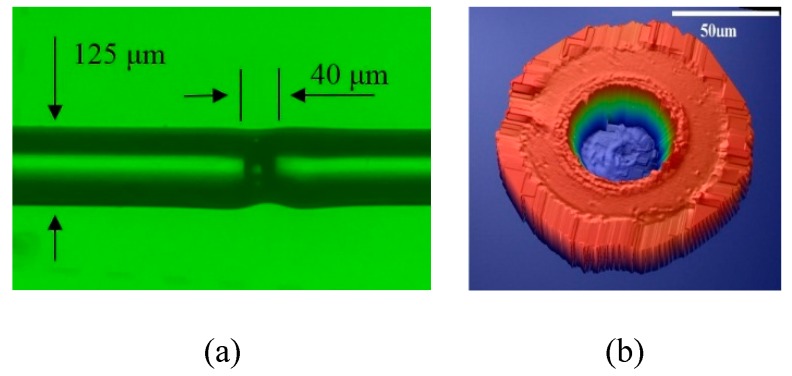
(**a**) Optical microscope photo of an FFPI strain gauge, and (**b**) cross-section view of the laser-etched Fabry–Pérot (FP) cavity.

Figure 4a shows a fabricated FFPI strain gauge using a standard multimode fiber (50/125 μm-graded index). A cross-section view of the laser-etched cavity is shown in Figure 4b, where the diameter and depth of the etched cavity are approximately 50 and 40 μm, respectively.

### 2.3. Spectrum Measurements

**Figure 5:**
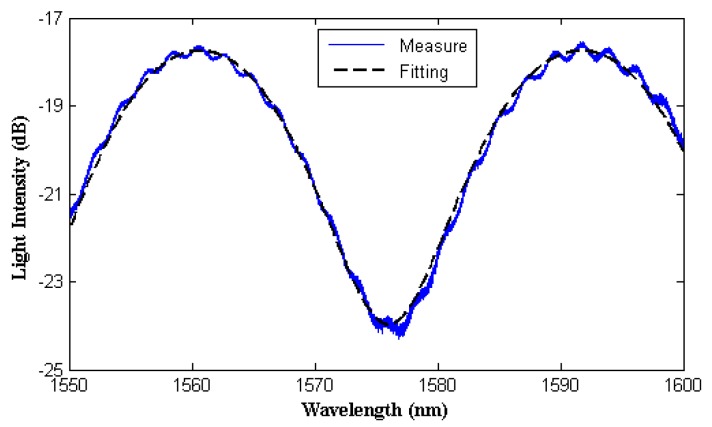
Measured and fitted reflection spectrum of an FFPI strain gauge.

Testing measurements of each FFPI strain gauge were carried out by employing an optical phase demodulator with an internal broadband light source. The reflection spectrum is shown in Figure 5, as well as the fitting using sinusoidal function. 

**Figure 6:**
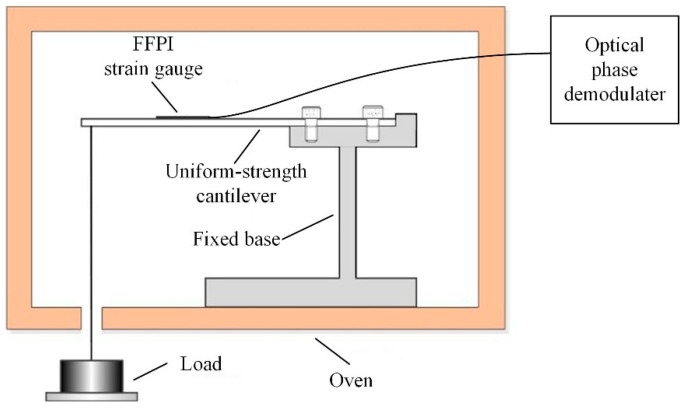
Measurement setup of the FFPI strain gauge.

**Figure 7:**
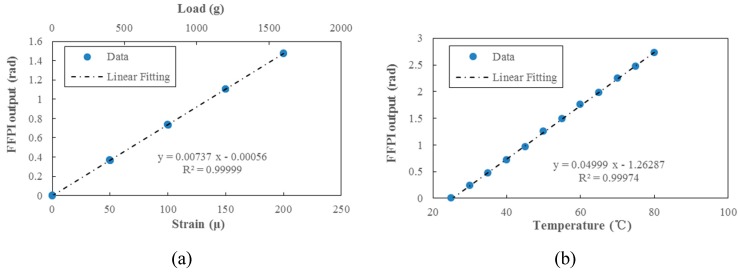
Results of (**a**) applied strain/load, and (**b**) temperature measurements of an FFPI strain gauge.

The FFPI strain gauge was installed on a uniform-strength cantilever to measure the applied strain response. The measurement set-up is shown in Figure 6. The cantilever was loaded with calibrated weights, to induce known changes in the cantilever’s surface strain (known changes in the cantilever surface strain were calculated from cantilever geometry and the material’s Young’s modulus). Figure 7a shows the measuring result for the dependence of the strain on the FFPI strain gauge’s shifted phase. It is found that a good linearity, with better than a 0.99999 linear correlation coefficient for the present sensor, can be obtained. The strain sensitivity (dϕ/d*ε*, change in phase shift/applied strain) of this gauge is estimated to be 0.0074 rad/με. Since the phase precision of our optical phase demodulator is 0.001 rad, it is expected that the minimum detectable strain alteration is about 0.135 με in our set-up.

The cantilever was then placed in an oven chamber which has a temperature accuracy of 0.1 °C. During the process of heating up, each sampling point was stabilized for 15 min; thus, the thermal distribution was homogeneous and there was no temperature gradient in the chamber. Figure 7b illustrates the temperature dependence of measured phase shift for the FFPI strain gauge. The temperature sensitivity dϕ/dT was measured to be 0.05 rad/°C. Thus, for the mounted FFPI strain gauge, a 1 °C temperature change generates an equivalent strain of 6.8 με, which means its strain sensitivity to temperature is 6.8με/°C.

FFPI itself has a minimal temperature strain cross sensitivity, due to the low thermal expansion coefficient of the fiber and the thermo-optical coefficient of the air cavity. However, since it is bonded onto the base material, which would have a big thermal expansion coefficient, the FFPI strain gauge has a much higher temperature sensitivity.

## 3. Force Balance Based on FFPI Strain Gauges

In a wind tunnel facility, the direct measurement of aerodynamic loads induced on the aircraft model are performed by a force balance. The balance is a precision-machined device that has strain gauges at strategic locations to measure the strain (i.e., deformations) due to applied forces and moments. Conventional force balances make use of resistive foil strain gauges to form active full Wheatstone bridges [26]. This section reports a novel balance based on FFPI strain gauges aimed at applications in hypersonic wind tunnels.

### 3.1. Hypersonic Wind Tunnel Set-Up

**Figure 8:**
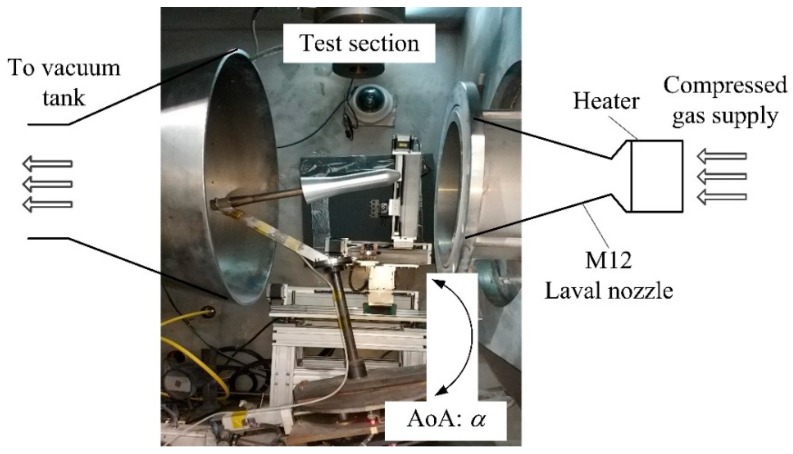
Schematic of the hypersonic wind tunnel set-up.

The force balance is aimed at measuring aerodynamic loads in the Φ 0.3-m low-density hypersonic wind tunnel (LDWT) in the Hypersonic Aerodynamics Institute (HAI), China Aerodynamics Research and Development Center (CARDC). Figure 8 shows the wind tunnel schematic set-up. The desired aerodynamic angle of attack (AoA) of the model can be set by a pitch apparatus. The tunnel expands high-pressure nitrogen gas through a convergent–divergent Laval nozzle to very low pressures. With pressure ratios of 10^−5^ to 10^−8^, it is possible to accelerate the test gas to high Mach numbers, M12 in this case, at low densities. Due to extreme expansion, the gas must be heated in advance to up to 600 K to avoid condensation. This results in heating of the model and, hence, the force balance located inside, requiring adequate temperature compensation.

### 3.2. Force Balance Configuration

**Figure 9:**
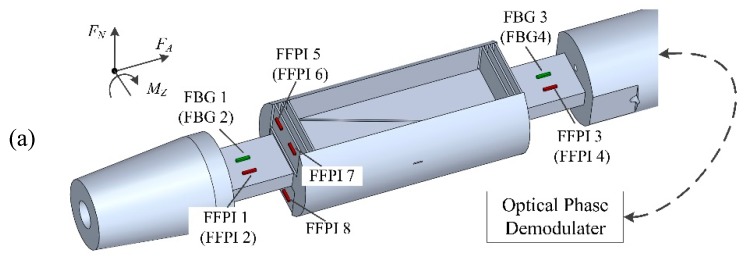

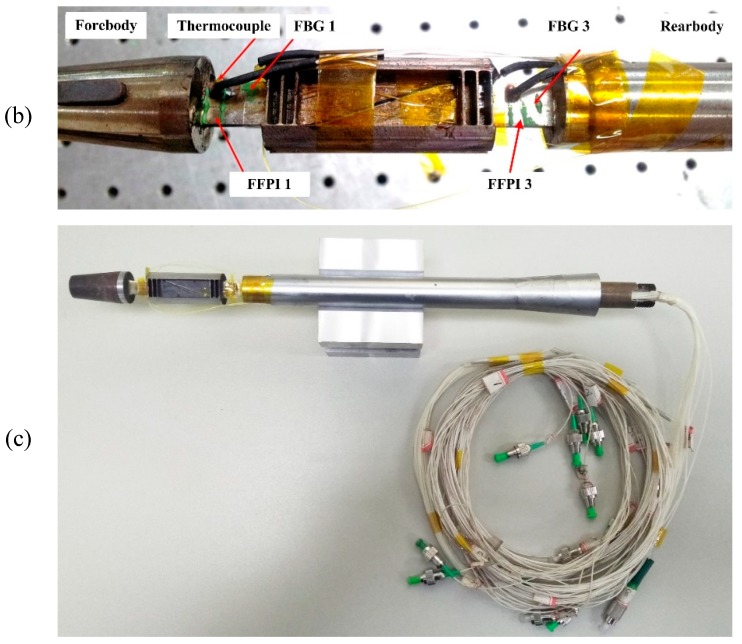
Force balance mounted with eight FFPI strain gauges and four fiber Bragg grating (FBG) temperature sensors: (**a**) design scheme, and (**b**) detailed and (**c**) overall photos of the balance.

For convenience, the total aerodynamic force vector is usually resolved into components. The body-oriented components are the axial force (*F_A_*), which is the force parallel to the vehicle axis, and the normal force (*F_N_*), which is the force perpendicular to the vehicle axis; the forces and pitching moment (*M_Z_*) are depicted in Figure 9a [27,28].

*F_A_*, *F_N_*, and *M_Z_* are measured with a three-component force balance, machined using high-quality F141 steel, with high mechanical performance, good machinability, and high corrosion resistance. To minimize the interaction between wiring and sensing structures, specific grooves and holes are designed in some areas of the balance. The separation of forces and moments was made possible by structural design of the balance and by the way the strain gauges were positioned. Each force or moment component was proportional to the strain applied on specific flexural elements. Strain gauges were arranged on the flexural elements to convert the strain (and, hence, the applied aerodynamic loads) to phase shifts, which are detected by an optical phase demodulator. Figure 9 shows the balance used in this work. The principle of the three-component load measurement principle is as follows:

*F_A_* component: By an inclined cut, the balance is separated into a model fixed part (forebody) and a sting fixed part (rearbody). These two parts are connected to each other by parallelogram flexure beams. These beams are able to withstand the loads of other components, but relatively flexible in the direction of axial load. Gauges FFPIs 5–8 are mounted on beams of the flexures.

*F_N_* and *M_Z_* components: The normal force *F_N_* and pitching moment *M_Z_* are measured by two rectangular side beams, which are symmetrically located on the front and rear sections with respect to the central axial section. Four gauges FFPIs 1–4 are installed on the side beams (FFPIs 2 and 4 in brackets means they are not shown in the figure, and are located symmetrically on the other side of the beam along the balance axial). The pitching moment *M_Z_* results in complete tension or compression in the side beams of equal strain magnitude; thus, the addition of the strains at the gauge position gives a signal proportional to the moment, while the subtraction of these strains results in the normal force *F_N_*.

**Figure 10:**
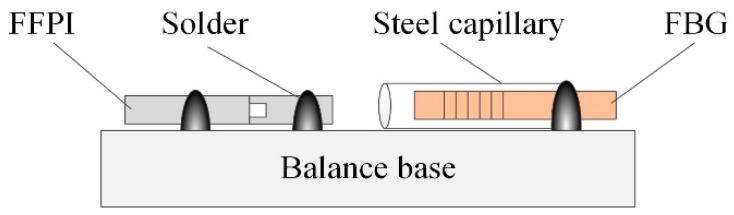
Schematic drawing of bonding techniques of FFPI and FBG sensors.

Four FBG temperature sensors are placed close to the FFPI strain gauges, with calibrated sensitivity of 0.01 nm/°C in Bragg wavelength shift. The FBG is encapsulated in a steel capillary to isolate any strain of the balance (see Figure 10 for the bonding techniques of FFPI and FBG sensors). These FBG sensors are used to monitor temperature changes during the operation of the balance, potentially correct temperature effects that are generated by the hypersonic hot flow, and compare the temperature compensation efficiency with the self-compensation method, which is proposed later in this work.

### 3.3. Temperature Self-Compensation of the Balance

For the mounted FFPI strain gauge, a 1 °C temperature change is measured to generate an equivalent strain of 6.8 με, calling temperature compensation for precise strain measurements. A traditional way to address this problem is to use a separate temperature sensor to obtain the temperature information and compensate for the temperature effect in the strain readings, which adds complexity to the measurement system. Furthermore, the strain sensing and temperature sensing happen at different locations, which would induce extra errors especially in a highly fluctuating temperature environment.

**Figure 11:**
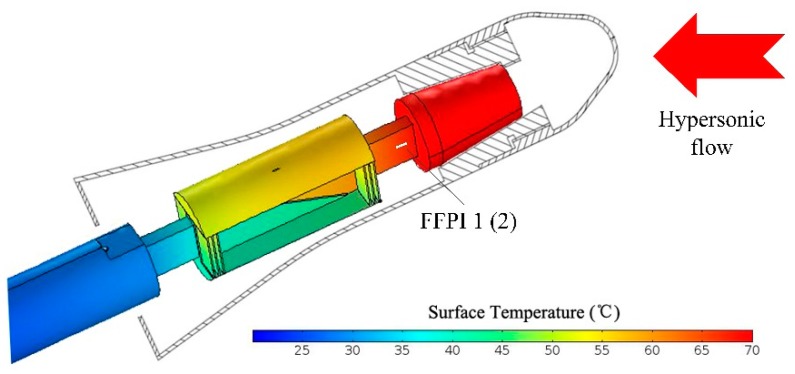
Temperature simulation of the balance, with an angle of attack (AoA) of 30°, and the tunnel model is assumed to be heated up to 70 °C.

**Figure 12:**
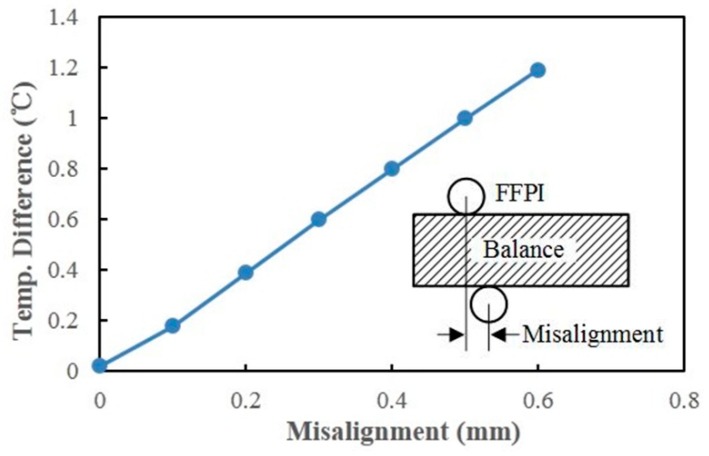
Simulated temperature difference between the positions of FFPIs 1 and 2, as a function of misalignment along the balance axial plane.

In consideration of good thermal conductivity of the balance material, a pair of FFPI gauges, located symmetrically along the balance axial, can be used to mitigate the drift caused by thermal expansion of the balance. Heat transfer simulations were carried out, using finite element analysis software COMSOL (5.3a, COMSOL Inc., Stockholm, Sweden), to verify the temperature distributions on the balance. Simulation parameters are shown in Table 1. Since the balance is operated in the Φ 0.3-m LDWT, where the static pressure is normally lower than 30 Pa, only heat transfer by conduction is considered in the present simulation. Assuming the tunnel model is set at an angle-of-attack (AOA) of 30° and is heated up to 70 °C, the simulated temperature distribution of the balance is shown in Figure 11. The temperature differences between FFPI 1 and FFPI 2, which are located on both sides of the balance beam symmetrically along the balance axial, are calculated as a function of misalignment, as shown in Figure 12. The gauge mounting tolerance is secured to within 0.2 mm in our process; thus, the temperature difference between these two gauges is assumed to below 0.4 °C, according to the present simulation.

Since the above simulation results imply that a pair of FFPI strain gauges mounted at symmetrical locations of the balance beam have similar temperature, they can be used to mitigate the drift caused by thermal expansion of the balance material. Under applied loads, the gauges in concave and convex positions of the balance beam experience near-equal blue (positive) and red (negative) shifts, respectively, due to bending of the beam, which can be used in temperature and strain discrimination.

The mechanical strain developed across the balance beam induces compressive and tensile strain in FFPI 1 and FFPI 2, respectively, and shifts the FFPI phase due to the thermo-optic effect. The shift in the FFPI phase due to strain (*ε*) and temperature changes (ΔT) is given by

(4) $$\Delta \Phi_{\mathrm{FP}1}=\alpha_{1}\Delta T+\beta_{1}\varepsilon ,$$

(5) $$\Delta \Phi_{\mathrm{FP}2}=\alpha_{2}\Delta T-\beta_{2}\varepsilon ,$$

where α relates to the coefficient of thermal expansion of silica, balance material, and thermo-optic coefficient of fiber, and β is a strain-optic coefficient, related to FP cavity depth and FFPI mounting length.

Adding Equations (4) and (5) isolates the sensor’s response to temperature change as

(6) $$\Delta T=k_{1}\left(\Delta \Phi_{\mathrm{FP}1}+k_{2}\Delta \Phi_{\mathrm{FP}2}\right),$$

where $k_{1}=\beta_{2}/\left(\alpha_{1}\beta_{2}+\alpha_{2}\beta_{1}\right)$ and $k_{2}=\beta_{1}/\beta_{2}$. Ideally, by choosing and mounting two identical FFPIs, $k_{1}$ and $k_{2}$ in Equation (6) can be assumed as 1/2 and 1 [29], respectively. However, the identical assumption is not easy to achieve practically; the mounted gauges should have different sensitivity and need to be calibrated. $k_{2}$ relates to the strain sensitivity of the gauges, and can be easily obtained from a simple loading experiment, while $k_{1}$ can be derived from temperature experiments.

Straightforwardly, the strain effect can be isolated from the difference between Equations (4) and (5). Note that ΔT can also be monitored from the FBG sensor placed close to the FFPI gauge. Both compensation efficiencies are experimentally examined and discussed in Section 4 and Section 5.

## 4. Results and Analysis

### 4.1. Static Calibration Accuracy

Direct and independent measurements of three aerodynamic loading components using the balance are generally impossible, since the signal is not only proportional to the measured component but also contains a small and complicated mixture of signals proportional to some or all of the other components, calling for calibration prior to wind tunnel applications. The calibration involves applying known loads to the balance, recording strain gauge readings at each force and moment combination, and calculating the applied loads from gauge readings. The detailed calibration method used in CARDC-HAI can be found in Reference [30]. The difference between any true load applied to the balance and the calculated load is the error of the calibration. It is often stated as a percentage of the corresponding component maximum calibrated load.

For the prototype FFPI strain gauge-based balance (labeled as “FFPI balance”), the gauge combination to every load component is sorted in Table 2, and Table 3 shows the calibration performances of the FFPI balance, as well as a traditional balance of the same mechanical structure using 1000 Ω foil resistive strain gauges (labeled as “FR balance”) for comparison. It can be seen that the accuracy of *F_N_* and *M_z_* components of the FFPI balance qualitatively agrees with that of the FR balance. The accuracy of *F_A_* component is poorer in both cases, which would be due to the mechanical complexity of this component and its relatively higher manufacturing tolerance.

### 4.2. Temperature Compensation during Oven Heating

**Figure 13:**
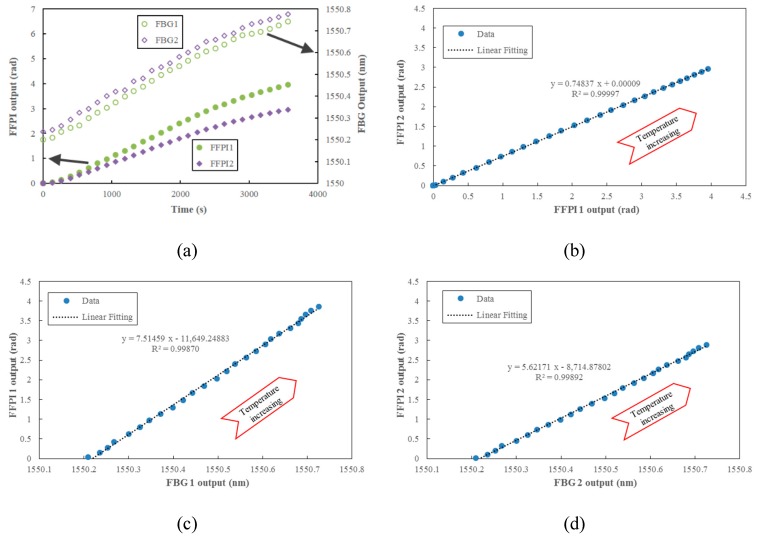
Thermal performances of the FFPIs and FBGs located on the forebody of the balance from room temperature (~25 °C) to 80 °C: temperature outputs with time (**a**), and relationships between different sensors (**b**–**d**). The higher linear correlation coefficient (*R^2^*) in (**b)**–(**d)** from linear fitting indicates a lower temperature fluctuation between the sensors.

To evaluate the possible drift of the zero output, the FFPI balance is placed in an oven chamber where the temperature is controlled to slowly increase from room temperature (~25 °C) to 80 °C, lasting one hour. Taking the FFPIs 1 and 2 and FBGs 1 and 2 located on the forebody of the balance as an example, all sensor outputs increased with temperature, as shown in Figure 13a. After replotting their outputs in Figure 13b–d, it can be seen that the output linearity correlation coefficient *R^2^* between FFPIs 1 and 2 is better than that between the FFPIs and the FBGs, indicating that the temperature difference between the FFPIs at symmetrical locations of the balance beam are much smaller, while there are higher temperature fluctuations between the FFPI and the FBG, even though they are located side by side.

### 4.3. Temperature Compensation during Hypersonic Wind Tunnel Runs

The measuring of aerodynamic loads on a hypersonic aircraft model is more challenging, as it involves working in a highly fluctuating temperature environment. In order to assess the effect of temperature compensation, the FFPI balance was placed in the wind tunnel with a fixed 10° AoA, for M12 gas blows. Since the AoA of the aircraft model is fixed, the aerodynamic load on it is expected to be stable.

**Figure 14:**
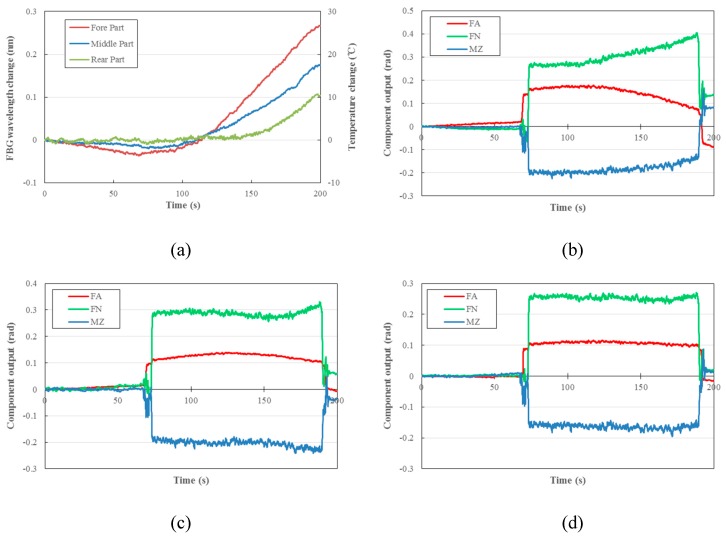
(**a**) Measured temperature changes in different parts of the balance according to FBG wavelength shifts, and (**b**) component outputs without temperature compensation, as well as those after (**c**) FBG compensation and (**d**) self-compensation.

Figure 14a shows the temperature changes of the fore, middle, and rear parts of the balance over a 200-s test period. It is evident, as shown in Figure 14b, that the balance exhibits some nonideal behaviors. The temperature drift issues are significant enough that the researchers are not confident in the collected data. The results after FBG and self-compensations are shown in Figure 14c,d respectively; it can be seen that the compensation efficiency by the latter one is better than that by the former one, since the components’ output is more stable due to the aerodynamic loads, and the temperature effect is much suppressed. The detailed thermal output of each component of the FFPI balance, after different temperature compensation treatments, is sorted in Table 4.

## 5. Discussion on Temperature Compensation

Due to the complexity of the temperature field of the force balance, the compensation effect is not only related to the temperature performance of the sensors itself, but also to strain caused by differential thermal expansion of the balance material. The FFPI sensors are tightly mounted on the surface of the balance metal beam, and the fiber’s heat capacity is very small; thus, they can quickly respond to any temperature changes of the balance. In the case of strictly ensuring the two FFPI gauges being installed in symmetrical positions, the temperature of the two gauges is assumed as the same. Therefore, in the wind tunnel experiment, the temperature output ratio *k* between the two FFPI gauges is similar to that obtained from oven heating; however, the thermal output of FFPIs would be high due to the thermal expansion of their base balance material. Meanwhile, since the FBG was encapsulated in a capillary to isolate any force/strain of the balance, its thermal output is much lower.

**Figure 15:**
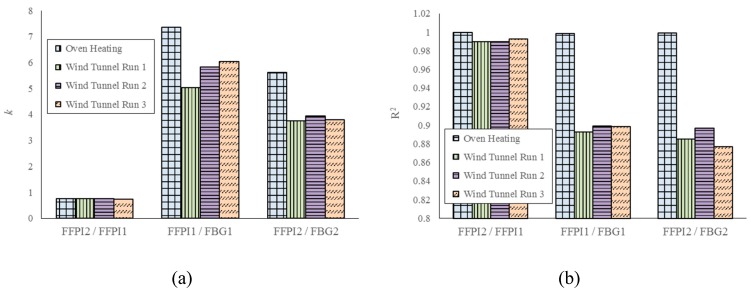
(**a**) Temperature output ratio *k*, and (**b**) linear correlation coefficient *R^2^* between different sensors, during oven heating and three wind tunnel runs.

Three wind tunnel runs were performed to further assess the effect of temperature compensation. From Figure 15a, one can find that the variation of temperature output ratio *k* between outputs of FFPI 1 and FFPI 2 is less than 1%, while that for FFPIs and adjacent FBGs is about 30%. The linear correlation coefficient *R^2^* between outputs of FFPIs and adjacent FBGs is about 0.9 during wind tunnel runs, much lower than that when slowly heating up in oven chamber.

The FBG sensors were encapsulated in a capillary, which results in, on the one hand, unavoidable friction between the FBG and capillary inner wall and, on the other hand, attenuation of heat transferred from the balance to the FBGs by the sandwiched capillary wall and gas medium. Therefore, the correction of temperature effects, calculated from measurements of point temperatures performed by FBGs, appeared as very unreliable and unrepeatable, as the FBGs insufficiently analyze the thermal variations occurring within the structure, especially for the hypersonic force balance designed to work in a highly fluctuating temperature environment.

The temperature self-compensation, nevertheless, can accurately calculate the temperature changes of the balance, and compensate for the temperature effect thereafter. Meanwhile, this solution does not require external temperature sensors, as both the gauge installation process and measurement system can be simplified.

## 6. Conclusions

This work reports an aerodynamic force balance aimed at applications in hypersonic wind tunnels, based on high-sensitivity, micromachined FFPI strain gauges. The FFPI strain gauges were fabricated with the assistance of 157-nm excimer laser processing, and the loaded strain was gauged by measuring the propagation phase shift of the reflected optical signal. The minimum detectable strain alteration was about 0.135 με in our set-up, and the strain and temperature sensitivity of the mounted FFPI strain gauge were about 0.0074 rad/με and 0.05 rad/°C, respectively. The accuracy of the proposed FFPI balance is better than 0.5% at full scale according to the static calibration.

Since the mounted FFPI strain gauge generates an equivalent strain of 6.8 με from a 1 °C temperature change, temperature compensation is necessary for the measurement of aerodynamic loads in a hypersonic wind tunnel, as it involves working in a highly fluctuating temperature environment. A self-compensation solution is proposed, using pairs of FFPI gauges located symmetrically along the balance axial to deduce the balance temperature change and mitigate the temperature effect. Separate FBG temperature sensors are also installed on the balance, and used for alternative temperature compensation. Both compensation effects were experimentally examined and the results show that, compared to FBG compensation, the self-compensation is more accurate for analyzing the thermal variations occurring within the balance structure and, therefore, more efficient for temperature compensation.

## Figures and Tables

**Table 1:** Simulation parameters of the balance.

Parameters	Symbol	Unit	Value
Heat capacity at constant pressure	*C_p_*	J/(kg∙K)	449
Density	*ρ*	kg/m^3^	8000
Thermal conductivity	k	W/(m∙K)	80.2
Young’s modulus	*E*	Pa	187 × 10^9^
Poisson’s ratio	*υ*	1	0.27
Coefficient of thermal expansion	*α*	K^−1^	10.7 × 10^−6^

**Table 2:** Strain gauge combinations for the three aerodynamic loading components. FFPI—all-fiber Fabry–Pérot interferometric strain gauge.

Component	Gauge Combination
*F_A_*	(FFPI 5 − FFPI 7) + (FFPI 6 − FFPI 8)
*F_N_*	(FFPI 1 − FFPI 2) − (FFPI 3 − FFPI 4)
*M_z_*	− (FFPI 1 − FFPI 2) − (FFPI 3 − FFPI 4)

**Table 3:** Calibration performances of the FFPI balance, compared with a traditional foil resistive (FR) balance.

Component	Design Load	Calibrated Accuracy	
		FFPI Balance	FR Balance
*F_A_*	12 N	0.455%	0.321%
*F_N_*	16 N	0.192%	0.162%
*M_z_*	0.48 N∙m	0.130%	0.094%

**Table 4:** Maximum thermal output of each component of the FFPI balance (unit: rad).

Component	Without Compensation	FBG Compensation	Self-Compensation
*F_A_*	0.13	0.04	0.01
*F_N_*	0.14	0.05	0.01
*M_z_*	0.07	0.04	0.01
